# Improved Plasmid-Based Inducible and Constitutive Gene Expression in *Corynebacterium glutamicum*

**DOI:** 10.3390/microorganisms9010204

**Published:** 2021-01-19

**Authors:** Nadja A. Henke, Irene Krahn, Volker F. Wendisch

**Affiliations:** Genetics of Prokaryotes, Faculty of Biology & CeBiTec, Bielefeld University, 33615 Bielefeld, Germany; n.henke@uni-bielefeld.de (N.A.H.); irene@cebitec.uni-bielefeld.de (I.K.)

**Keywords:** expression vector, *Corynebacterium glutamicum*, overexpression, promoter, origin of replication

## Abstract

*Corynebacterium glutamicum* has been safely used in white biotechnology for the last 60 years and the portfolio of new pathways and products is increasing rapidly. Hence, expression vectors play a central role in discovering endogenous gene functions and in establishing heterologous gene expression. In this work, new expression vectors were designed based on two strategies: (i) a library screening of constitutive native and synthetic promoters and (ii) an increase of the plasmid copy number. Both strategies were combined and resulted in a very strong expression and overproduction of the fluorescence protein GfpUV. As a second test case, the improved vector for constitutive expression was used to overexpress the endogenous xylulokinase gene *xylB* in a synthetic operon with xylose isomerase gene *xylA* from *Xanthomonas campestris*. The xylose isomerase activity in crude extracts was increased by about three-fold as compared to that of the parental vector. In terms of application, the improved vector for constitutive *xylA* and *xylB* expression was used for production of the *N*-methylated amino acid sarcosine from monomethylamine, acetate, and xylose. As a consequence, the volumetric productivity of sarcosine production was 50% higher as compared to that of the strain carrying the parental vector.

## 1. Introduction

*C. glutamicum* was discovered in the 1960s as a natural L-glutamate producer [1]. Since then, both its genetic toolbox [2] and its number of heterologous pathways [3,4] have been extended. On the one side, production of value-added compounds such as amino acids [5,6], organic acids [7,8], and terpenoids [9,10] has been established. Recently, the production of sarcosine (*N*-methylglycine) was enabled by overexpression of the imine reductase DpkA from *Pseudomonas putida* in a glyoxylate-overproducing *C. glutamicum* strain by providing monomethylamine as the methyl-donor [11]. On the other side, several approaches were followed in order to establish a flexible feedstock concept that allows *C. glutamicum* production strains to grow/produce on the basis of a variety of non-food competitive substrates such as industrial or agricultural/aquatic side streams [12]. The access to glycerol, the stoichiometric byproduct of biodiesel production, was enabled and applied to various production strains [13,14]. Moreover, recent attempts have aimed to establish the methylotrophy in *C. glutamicum* for methanol utilization [15]. Besides the sugar polymers starch [16] and cellulose [17], the pentose sugars xylose and arabinose [18,19] that derive from hemicellulose can be used as alternative substrates for a variety of high-value products including the fragrance compound patchoulol [20] and the potential antipsychotic compound sarcosine [11].

Many of these production strain-engineering efforts rely on gene expression vectors, which represent a powerful tool not only for metabolic engineering, but also for in depth analysis of basic metabolic principles in *C. glutamicum* that facilitate the development of new metabolic engineering strategies. For *C. glutamicum,* a wide range of different expression vectors have been used in research: inducible expression vectors, constitutive expression vectors [21], dual-expression vectors [22,23], promoter-probe vectors [24], and suicide-vectors for homologous recombination [25]. Most of the expression vectors used today for *C. glutamicum* are based on the origins of replication pBL1, pGA1, pCG1, and pHM1519 that derive from natural plasmids of corynebacteria [26].

Among many others, pVWEx1 and pECXT99A represent well-established expression vectors for *C. glutamicum* that use the P*tac*/P*trc* promoter and carry a *lacI* gene allowing for IPTG-inducible gene expression [27,28]. These IPTG-inducible expression systems make use of the Lac repressor from *E. coli* since *C. glutamicum* lacks a homolog and they provide *lac* operator(s) [29]. Replication of pVWEx1 in *C. glutamicum* relies on the pHM1519 replicon. Recently, it was shown that a mutation in the initiator protein RepA improved the plasmid copy number of pHM1519 origin to around 800 copies per cell [30].

The promoter structure and consensus sequence have been intensively examined [31]. In addition, the assignment of sigma factors of the RNA polymerase to the respective promoters [32] paved the way to the identification of their regulatory networks [33,34,35]. The promoters that are depending on the house-keeping sigma factor SigA or the alternative sigma factor SigB are suitable for application in metabolic engineering [35,36]. Therefore, the choice of interesting promoters as genetic elements in vector design also relies on the respective sigma factor that facilitates promoter recognition. Libraries of natural and synthetic promoters have been screened and identified, e.g., the synthetic promoter H36 was described to be 16-fold stronger than the P*trc* promoter [37] and a synthetic promoter comprising the −10 consensus sequence TAnnnT from *C. glutamicum* and the −35 motif TTGACA supported very high transcription [38].

In this work, we assessed the improvement of plasmid copy number and the choice of strong promoters alone or in combination for plasmid-borne target gene expression. In addition to scoring fluorescent reporter gene expression, we applied the gained insight to improve production of the *N*-methylated amino acid sarcosine from monomethylamine, acetate, and xylose.

## 2. Materials and Methods

### 2.1. Bacterial Strains and Growth Conditions

Strains and plasmids used in this study are listed in Table 1. Newly constructed vectors were evaluated to be functional in *C. glutamicum* WT. Chemicals were delivered by Carl Roth (Karlsruhe, Germany) if not stated differently. Precultures of *C. glutamicum* strains were grown in complex medium Luria broth (LB) (5 g/L yeast extract, 10 g/L tryptone, 10 g/L NaCl) or brain heart infusion (BHI) (37 g/L) supplemented with 50 mL 2% glucose overnight in 500 mL baffled flasks. Main cultures were grown in CGXII minimal medium (20 g/L (NH_4_)_2_SO_4_, 5 g/L urea, 1 g/L KH_2_PO_4_, 1 g/L K_2_HPO_4_, 42 g/L MOPS, 0.25 g/L MgSO_4_ × 7 H_2_O, 10 mg/L CaCl_2_, 10 mg/L FeSO_4_ × 7H_2_O, 10 mg/L MnSO_4_ × H_2_O, 1 mg/L ZnSO_4_ × 7H_2_O, 0.2 mg/L CuSO_4_, 0.02 mg/L NiCl_2_ × 6H_2_O, 0.2 mg/L biotin (Sigma-Aldrich, Taufkirchen, Germany), and 30 mg/L protocatechuate (VWR, Darmstadt, Germany)) supplemented with the named carbon source after washing in TN-buffer (10 mM Tric-HCl (pH 6.3), 10 mM NaCl). Sarcosine production was performed with 12 g/L xylose and 20 g/L potassium acetate as carbon sources and a reduced concentration of nitrogen (2 g/L (NH_4_)_2_SO_4_ and 0.5 g/L urea) in the presence of 3.1 g/L monomethylamine (MMA) (TCI, Eschborn, Germany). Main cultures were inoculated to an initial OD_600 nm_ of 1 using a Shimadzu UV-1202 spectrophotometer (Duisburg, Germany). To achieve the high aeration required for aerobic cultures of *C. glutamicum*, cultivations were performed in 50 mL of culture medium in 500 mL baffled shake flasks at 120 rpm, or alternatively in 1 mL culture medium in the Biolector^®^ flowerplate system (m2p-labs GmbH, Baesweiler, Germany) at 1100 rpm at 30 °C. *E. coli* DH5α cells were cultivated at 37 °C in LB medium. Tetracycline, kanamycin, and spectinomycin (VWR, Darmstadt, Germany) were added if appropriate in concentrations of 5, 25, and 100 µg mL^−1^. 

### 2.2. Construction of New Expression Vectors

The new expression plasmids were constructed in *E. coli* DH5α. First, target promoters or genes were amplified by a high-fidelity PCR (All-in HiFi, highQu, Kraichtal, Germany) and cloned into digested or backbone-amplified expression vectors by Gibson-Assembly [44]. The oligonucleotides are listed in Table 2 and were delivered by Metabion (Planegg/Steinkirchen, Germany). The PCR amplificates were purified with a PCR and gel extraction kit (Macherey-Nagel, Düren, Germany). *E. coli* DH5α-competent cells were prepared with CaCl_2_ and transformed via heat shock. Recombinant clones were screened by colony-PCR and plasmids were isolated with a miniprep kit (GeneJET, Thermo Fisher Scientific, Schwerte, Germany). New expression vectors were confirmed by sequencing. *C. glutamicum* cells were transformed by electroporation as described elsewhere [45].

### 2.3. Cloning of pECXT99A and pVWEx1-Based Expression Vectors

For construction of the pECXT99A-based expression vectors, pECXT99A was digested with NcoI and NdeI (Thermo Scientific Fisher, Schwerte, Germany) and dephosphorylated (Antarctic phosphatase, New England Biolabs, Frankfurt, Germany). Target promoters were amplified using the oligonucleotides as follows: P*tuf* (cg0587): HN12 + HN13; P*gapA* (cg1791): HN14 + HN15; P*ilvC* (cg1437): HN16 + HN17; P*sodA* (cg3237): HN30 + HN31; P*pgk* (cg1790): HN97 + HN98; P*45*: HA02 + HA03; P*H36*: HA04 + HA05; P*syn*: HA06 + HA07. The native promoters were amplified from the genomic DNA of *C. glutamicum* WT. The synthetic promoters P*syn* (5′-TTGACATTAATTTGAATCTGTGTTATAATGGTTC-3′), P*H36* (5′-CAAAAGCTGGGTACCTCTATCTGGTGCCCTAAACGGGGGAATATTAACGGGCCCAGGGTG.

GTCGCACCTTGGTTGGTAGGAGTAGCATG-3′), and P*45* (5′-TTGGTCAGGGATTTTTTCCCGAGGGCACTAATTTTGCTAAAGTAAGTGACGAAGAAGTTC-3′) were synthesized or inserted by oligonucleotides in the case of P*45*. All constructs were checked by sequencing. Newly constructed expression vectors were digested with BamHI and the fluorescence reporter gene *gfpUV* (HN49 + HN50) was cloned via Gibson-Assembly.

For construction of the pVWEx4 expression vectors, a site-directed mutagenesis in the *repA* gene was performed on pVWEx1 for an increased plasmid copy number. Site-directed mutagenesis was conducted via plasmid backbone amplification (HA36 + HA37) with *Pfu* Turbo DNA Polymerase. The vector pVWEx6 was constructed via digestion of pVWEx4 with KpnI and MauBI and insertion of the P*syn* promoter including a *lac* operator motif (N105 + N106).

### 2.4. Fluorescence Analysis

GfpUV fluorescence was measured on a FACS GalliosTM (Beckmann Coulter GmbH, Krefeld, Germany) with 405 nm excitation from a blue solid-state laser. Forward-scatter characteristics (FSC) and side-scatter characteristics (SSC) were monitored as small- and large-angle scatters of the 405 nm laser. Fluorescence was detected using a band-pass filter (500/50 nm). The produced GfpUV protein possesses characteristic emission at 509 nm with an excitation wavelength of 385 nm. *C. glutamicum* WT harboring the newly constructed plasmids or the control plasmid were harvested in stationary growth, washed once in phosphate-buffered saline, and the optical density was adjusted to OD_600nm_ ~0.1. *C. glutamicum* WT was used to determine autofluorescence. Median fluorescence intensities of 20,000 cells were calculated from each culture.

### 2.5. SDS-PAGE

*C. glutamicum* WT cells carrying the newly constructed plasmids were grown at 30 °C in 1 mL in Biolector^®^ flowerplate system (m2p-labs GmbH, Baesweiler, Germany). Cells were harvested by centrifugation and stored at −20 °C. For cell extract preparation, thawed cells were re-suspended in KPI buffer (100 mM disodium hydrogenphosphate and 100 mM sodium dihydrogenphosphate with pH 6.9). Cells were disrupted by ultrasonification using Hielscher UP200S2 (Teltow, Germany) with an amplitude of 60% and a pulsing cycle of 0.5 (power discharge 0.5 s, pause 0.5 s) for 9 min. After ultracentrifugation (1 h, 45,000× *g*, 4 °C) the protein concentration was determined with Bradford Reagent (Sigma-Aldrich, Germany). Then, a 10 µg protein sample was mixed with Lämmli-Buffer and loaded on an SDS-PAGE, comprising a 4% stacking gel and 10% running gel. Gels were initially run at 50 V and then at 100 V. Protein gels were stained in 0.1% Coomassie blue solution (30% methanol; 10% acetic acid).

### 2.6. Enzyme Assay for Xylose Isomerase XylA

Cells of the strains WT (pECXT99A-*xylAB*) and WT (pECXT_P*syn*-*xylAB*) were inoculated from a fresh LB overnight culture into a LB main culture with antibiotics and IPTG (1 mM) if applicable. Cultures were inoculated with an initial OD_600 nm_ of 0.6 and grown until OD_600 nm_ was 4. All cells from each culture were harvested at 4 °C and stored at −20 °C till further use. Cells were washed in Tris-HCl Buffer (100 mM pH 7.5) and resuspended in 2 mL buffer prior to ultrasonification using Hielscher UP200S2 (Teltow, Germany) with an amplitude of 60% and a pulsing cycle of 0.5 (power discharge 0.5 s, pause 0.5 s) for 9 min. After ultracentrifugation (1 h, 45,000× *g*, 4 °C) the protein concentration was determined with Bradford Reagent (Sigma-Aldrich, Germany). Enzymatic activity of XylA was measured from the raw extract in a total volume of 1 mL at 30 °C: 100 mM Tris-HCl pH 7.5, 10 mM MgCl_2_, 0.17 mM NADH, and 1 U sorbitol dehydrogenase. Then, 200 µL D-xylose (2.5 M) was added and absorption was measured for an additional 3 min using a Shimadzu UV-1650 PC photometer (Shimadzu, Duisburg, Germany). The assay was performed with three appropriate protein concentrations each, applying 5/10/15 µL of crude extracts from strains WT (pECXT99A-*xylAB*) and WT (pECXT_P*syn*-*xylAB*) with protein concentrations of 5.8 mg/mL and 1.72 mg/mL, respectively. Specific activities were calculated as µmol min^−1^ (mg protein)^−1^, defined as one unit (U/mg).

### 2.7. Sarcosine Quantification

Sarcosine quantification was performed from the clear supernatant of culture samples. Samples were derivatized with 9-fluorenylmethyl chlorocarbonate (FMOC) as decribed elsewhere [46]. Identification and separation was performed on a reversed phase HPLC. The column system consisted of a precolumn (LiChrospher 100 RP18 EC-5, 40 × 4 mm) and a main column (LiChrospher 100 RP18 EC-5, 125 × 4 mm) (CS Chromatographie Service GmbH, Langerwehe, Germany). Sodium acetate (50 mM pH 4.2) (A) and acetonitrile (B) were used as the mobile phases. A gradient with a flow rate of 1.2 mL min^−1^ was used as follows: 0 min 38% B; 10 min 38% B; 17 min 57% B; 19 min 76% B; 20 min 76% B, and 23 min 38% B. Detection of the fluorescent derivatives was performed by a fluorescence detector (FLD G1321A, 1200 series, Agilent Technologies, Ratingen, Germany) with an excitation wavelength of 263 nm and emission wavelength of 310 nm. Calibration was conducted with a sarcosine standard (Sigma-Aldrich, Steinheim, Germany). L-proline was used as an internal standard.

## 3. Results

### 3.1. Screening of Strong Constitutive Promoters in the pECXT99A-Based Vector System

**Figure 1:**
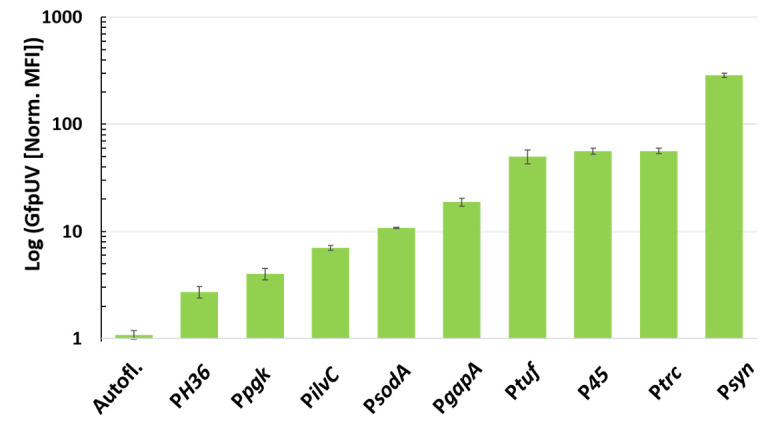
Fluorescence reporter assay of a promoter library in the pECXT99A plasmid background in *C. glutamicum* WT. Median GfpUV fluorescence intensities were normalized to an autofluorescent control. Mean values and standard deviations of biological triplicates are shown. Measurements were performed 18 h after inoculation in CGXII medium with 4% glucose. Endogenous promoters: P*pgk*, P*ilvC*, P*sodA*, P*gapA*, and P*tuf*. Synthetic promoters: P*H36*, P*45*, and P*syn*. Reference: the IPTG-inducible promoter P*trc* induced with 1 mM IPTG.

A selection of native and synthetic promoters was cloned in the pECXT99A-backbone (lacking the *lacI^q^* gene to allow for constitutive expression) in order to test the different promoter strengths using the promoterless reporter gene *gfpUV*. The promoters from the endogenous genes *tuf* (cg0587), *ilvC* (cg1437), *sodA* (cg3237), *gapA* (cg1791), and *pgk* (cg1790) were amplified from the genome of *C. glutamicum* WT. The synthetic promoters P*H36* and P*syn* were generated by gene synthesis, whereas P*45* was generated by oligonucleotide insertion. GfpUV reporter fluorescence in *C. glutamicum* WT carrying the plasmids was monitored 18 h after inoculation in CGXII with 4% glucose without any inducer. The strain carrying the parental plasmid pECXT99A-*gfpUV* (with *lacIq*) was chosen as a reference (cultured in the presence of 1 mM IPTG), as this vector served as the starting point for the cloning of the promoter library. All tested promoters showed activity and, thus, allowed for constitutive gene expression without the need to add an inducer to the medium (Figure 1). The synthetic promoter P*H36* (norm. MFI: 2.7 ± 13%) and the endogenous promoters P*pgk* (norm. MFI: 4.0 ± 12%) and P*ilvC* (norm. MFI: 7.0 ± 5%) showed significant expression in comparison to that of the autofluorescence (norm. MFI: 1.0 ± 10%). However, the expression was relatively low in comparison to that of the other vectors. The endogenous promoters P*sodA* (norm. MFI: 10.8 ± 1%), P*gapA* (norm. MFI: 19 ± 9%), and P*tuf* (norm. MFI: 50 ± 15%) showed increasing expression levels with the latter one showing comparable expression values to that of the synthetic P*45* (norm. MFI: 56 ± 7%). Thus, *gfpUV* expression from the constitutive P*45* was as strong as IPTG-induced *gfpUV* expression from P*trc* in pECXT99A (with *lacIq* and P*trc*; norm. MFI: 56 ± 6%). Notably, *gfpUV* expression from the synthetic promoter P*syn* exceeded this level by about a factor of five (norm. MFI: 286 ± 4%). Thus, the newly constructed pECXT_P*syn* showed the highest *gfpUV* expression of the compared vectors (Figure 1).

### 3.2. Improving Plasmid-Borne Inducible Gene Expression in C. glutamicum by Combination of a Stronger Promoter with Increased Plasmid Copy Number

**Figure 2:**
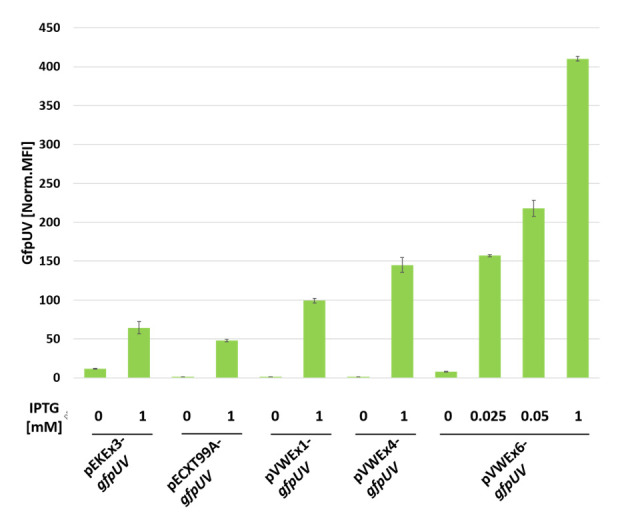
Fluorescence reporter assay of conventional and newly constructed IPTG-inducible expression vectors in *C. glutamicum* WT. Vector pECXT99A-*gfpUV* expressed *gfpUV* from P*trc*, while P*tac* was used for vectors pEKEx3-*gfpUV,* pVWEx1-*gfpUV,* pVWEx4-*gfpUV,* and pVWEx6-*gfpUV*. Median GfpUV fluorescence intensities were normalized to an autofluorescent control. Mean values and standard deviations of biological triplicates are shown. Measurements were performed 20 h after inoculation in CGXII medium with 4% glucose. Newly constructed vectors: pVWEx4 and pVWEx6. Reference vectors: pEKEx3, pECXT99A, and pVWEx1. Concentration of the inductor IPTG is given in mM.

**Figure 3:**
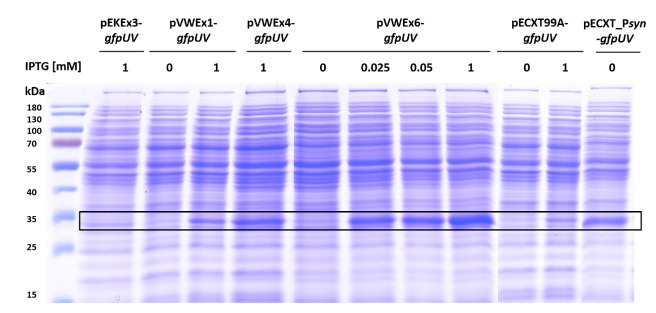
SDS-PAGE for comparison of GfpUV (27 kDa) protein abundance based on different expression vectors in *C. glutamicum* WT. 10 µg of crude extracts from the main cultivation in CGXII with 4% glucose was loaded on a 10% SDS-PAGE. Pre-stained protein ladder (26616 from Thermo Scientific) was used as reference standard. Concentration of the inductor IPTG is given in mM.

First, the properties of the commonly used IPTG-inducible expression vectors pEKEx3, pVWEx1, and pECXT99A were compared with regard to *gfpUV* gene expression by FACS analysis (Figure 2) and protein abundance by SDS-PAGE (Figure 3). Induction factors were determined from reporter fluorescence measured in the absence and presence of 1 mM IPTG. With vector pEKEx3-*gfpUV,* a maximal norm. MFI of 64 ± 13% was reached, but expression was leaky, thus, an induction factor of only 6 resulted (Figure 2). The vector pECXT99A*-gfpUV* supported a maximal norm. MFI of 48 ± 3% with an induction factor of 47. Expression from pVWEx1*-gfpUV* without IPTG was as tight as that observed for pECXT99A-*gfpUV*. With higher maximal norm. MFI of 99 ± 3% an induction factor of 76 was observed with pVWEx1-*gfpUV*. SDS-PAGE revealed lower GfpUV protein abundance for pEKEx3-*gfpUV* as compared to that of pECXT99A*-gfpUV*, which was highest among the three vectors for pVWEx1-*gfpUV* (Figure 3).

Next, we sought to improve inducible gene expression vector pVWEx1, which possesses the origin of replication pHM1519. It has been show that pHM1519-based vectors are maintained at plasmid copy numbers of approximately 140 in *C. glutamicum* cells [47]. Importantly, it has recently been shown that the copy number of vectors with a pHM1519 replicon can be increased about 5-fold to about 800 [30]. This finding prompted us to increase the plasmid copy number of pVWEx1. Therefore, the *repA* gene encoding an initiator protein for the pHM1519 replicon was mutated (base transition from G to A at nucleotide 1286) to exchange Gly at position 429 of RepA protein by Glu via site-directed mutagenesis, which was designated as *copA1* [30]. The resulting vector based on pVWEx1 with the mutation RepA^G429D^ was named pVWEx4. FACS analysis revealed an approximately 1.5-fold increased IPTG-induced *gfpUV* gene expression using vector pVWEx4-*gfpUV* as compared to that using pVWEx1-*gfpUV* (norm. MFI of 145 ± 7% as compared to 99 ± 3%). Since expression without IPTG was almost as tight for pVWEx4 as for pVWEx1, the induction factor with pVWEx4 was higher than that with pVWEx1 (121 as compared to 76). Thus, introduction of the *copA1* mutation into the *repA* gene increased the plasmid copy number as expected, and allowed for a very strong and more than 100-fold inducible target gene expression.

Third, since P*trc* was weaker than P*syn* (Figure 1), we hypothesized that replacing P*trc* in plasmid pVWEx4 by the stronger P*syn*, while maintaining the *lacIq* and *lac* operator sequences for IPTG-inducible gene expression, would lead to increased target gene expression that could be modulated by titrating IPTG concentrations. The resulting vector was named pVWEx6. This vector allowed for an approximately 50-fold induction with 1 mM IPTG, leading to a maximal norm. MFI of 410 ± 1% (Figure 2 and Table 3).

Moreover, we sought to find a lower IPTG concentration for pVWEx6 that would achieve comparable expression as that of the fully induced pVWEx1, pEXCT99A, and/or pEKEx3. Our choice of 0.025 mM and 0.050 mM IPTG as intermediate IPTG concentrations was guided by prior experience with vectors pVWEx1 and pEKEx3 for expression of genes for membrane proteins or transporter proteins [48,49,50]. Overexpression of genes for membrane proteins or transporter proteins proved difficult as too high levels reduced growth, probably by altering membrane integrity. However, with 0.025 mM or 0.050 mM IPTG as the intermediate IPTG concentrations, functional overexpression of genes coding for membrane proteins or transporter proteins was achieved without observed growth impairment [48,49,50]. Here, intermediate GfpUV fluorescence was observed with 0.025 and 0.05 mM IPTG. SDS-PAGE analysis of GfpUV reporter protein abundance (Figure 3) confirmed the FACS results of very strong expression (Figure 2). In fact, the IPTG-induction factor was as high as those obtained with pVWEx1 and pECXT99A, while the fully induced expression strength supported by pVWEx6 was one magnitude higher than those achieved with pEKEx3, pECXT99A, and pVWEx1.

### 3.3. Fast Production of Sarcosine from Xylose by Application of the Newly Constructed pECXT_Psyn

**Figure 4:**
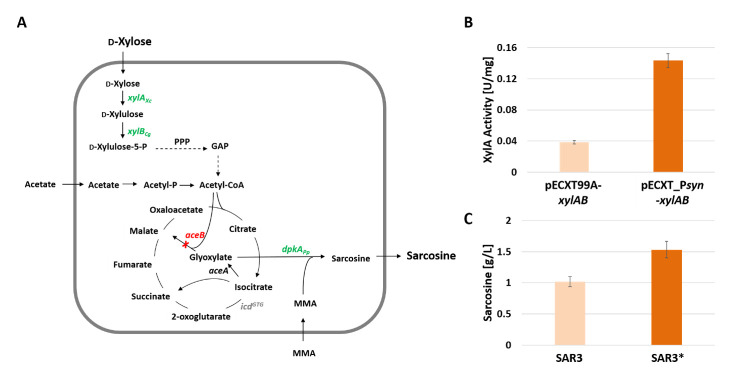
Schematic representation of sarcosine production by recombinant *C. glutamicum* (**A**), Xylose isomerase (XylA) activities in crude extracts measured in triplicates (**B**), and sarcosine titers produced by *C. glutamicum* strains SAR3 and SAR3* measured 20 h after inoculation from triplicates (**C**). XylA: xylose isomerase; XylB: xylulose kinase; PPP: pentose phosphate pathway; GAP: glyceraldehyde 3-phosphate; AceB: malate synthase; AceA: isocitrate lyase; DpkA: imine reductase; Icd: isocitrate dehydrogenase; MMA: monomethylamine.

As an application test, sarcosine production by *C. glutamicum* was chosen (Figure 4A). *C. glutamicum* was engineered to produce the *N*-methylated amino acid sarcosine in the enzyme reaction catalyzed by the *Pseudomonas putida* KT2440 imine reductase DpkA using glyoxylate as the 2-oxo acid substrate and monomethylamine as the *N*-alkyl donor [11]. To enable xylose utilization, the WT carrying malate synthase gene deletion Δ*aceB*, a start codon exchange from ATG to GTG for isocitrate dehydrogenase gene *icd* and the vector pVWEx1-*dpkA*_RBSopt for IPTG-inducible expression of *dpkA,* was transformed with pECXT99A-*xylAB* as the second vector. Notably, the resulting *C. glutamicum* strain SAR3 produced sarcosine with higher yield coefficients than that with glucose, however, the volumetric productivity was low due to slow growth with xylose [11]. To test if stronger expression of *xylAB* using the newly constructed vector pECXT99A_P*syn*-*xylAB* accelerates xylose-based sarcosine production, strain SAR3* that carried pECXT99A_P*syn*-*xylAB* instead of pECXT99A-*xylAB* was constructed (Figure 4A).

First, to score xylose catabolism an enzyme assay of the heterologous xylose isomerase XylA was performed. To this end, crude extracts of *C. glutamicum* WT carrying either pECXT99A_P*syn*-*xylAB* or pECXT99A-*xylAB* were prepared and assayed spectrophotometrically for XylA activity (Figure 4B). A more than 3-fold increase in the specific XylA activity indicated that pECXT99A_P*syn*-*xylAB* provided higher *xylAB* expression than pECXT99A-*xylAB* (Figure 4B). Next, sarcosine production by strains SAR3 and SAR3* was compared in media containing monomethylamine, potassium acetate, and xylose. When grown in the presence of acetate, *C. glutamicum* is known to carry a high flux from acetyl-CoA into the TCA cycle and to glyoxylate [7,51] (Figure 4A). This is due to transcriptional regulation of the isocitrate lyase gene *aceA* (and malate synthase gene *aceB* that is deleted in SAR3) by the transcriptional activator RamA and the transcriptional repressor RamB [52,53]. Xylose served as the primary carbon and energy source for growth under these conditions. Growth of SAR3* was 1.3-fold faster than growth of SAR3 (growth rates of 0.13 ± 0.01 h^−1^ as compared to 0.10 ± 0.01 h^−1^, respectively). Importantly, after 20 h, sarcosine accumulated to titers of 1.0 ± 0.08 g L^−1^ for SAR3 and 1.5 ± 0.13 g L^−1^ for SAR3* (Figure 4C). As a consequence, the volumetric productivity was improved by 50% (0.077 g L^−1^ h^−1^ for SAR3*) as compared to SAR3 (0.051 g L^−1^ h^−1^). Thus, pECXT99A_P*syn*-*xylAB* proved useful to improve the xylose-based sarcosine production by recombinant *C. glutamicum*.

## 4. Discussion

Expression vectors play a crucial role in basic research to investigate endogenous metabolism and to establish microbial bio-production by metabolic engineering. However, the development and/or optimization of expression systems is rarely the focus of scientific work. In this study, we focused on the optimization of the two well-established expression vectors pECXT99A and pVWEx1 by application of a strong promoter and by an increased plasmid copy number, respectively. Due to different *C. glutamicum* origins of replication and antibiotic resistance markers (Table 3), these vectors can co-exist: pEKEx3 plus pECXT99A or pECXT_P*syn* plus pVWEx1 or pVWEx4 or pVWEx6.

The expression level of a vector system is dependent on a wide range of aspects that affect either transcription or translation of an overexpressed gene or the protein stability itself. For example, plasmid-driven gene expression relies on the number of gene copies (plasmid-copy number) and the promoter strength. Both aspects were part of this work. In addition, the translational efficiency of target genes may be optimized by changing the translational start codon [54] or by changing ribosome binding sites as a trade-off between an optimal RBS (consensus *C. glutamicum*: 5′-GAAAGGAGG-3′) and the prevention of secondary mRNA structures [55,56,57]. Independent of vector characteristics, the target gene (and gene product) may be optimized. Protein stability can be improved either by adding tags, by construction of fusion enzymes [58,59], or by truncations to improve protein solubility [60,61] while taking into account dimerization domains, catalytic centers, and cofactor binding sites [62]. If their termini are freely accessible, membrane proteins may be fused as shown in the fusion of the cytosolic C terminus of CrtZ to the cytosolic N terminus of CrtW, which resulted in improved astaxanthin production by *C. glutamicum* [10]. The improved vectors developed here (pVWEx4, pVWEx6, and pECXT99A_P*syn*) add to the toolbox available to microbiologists studying the physiology of *C. glutamicum* as well as for metabolic engineering in strain and process development. Moreover, the expression characteristics obtained in comparative fluorescence reporter gene expression experiments will guide the choice of vectors according to the task of interest.

To compare plasmid-borne target gene expression, the endogenous promoters of the genes *pgk*, *ilvC*, *sodA*, *gapA,* and *tuf* were chosen in this study. The promoters of the glycolytic genes *pgk* [63] and *gapA* [64], encoding phosphoglycerate kinase and glyceraldehyde 3-phosphate dehydrogenase, respectively, as well as of the superoxide dismutase gene *sodA* [65], the ketol-acid reductoisomerase gene *ilvC* [65], and the transcription elongation factor gene *tuf* [66] have already been described as strong constitutive promoters. In a different vector background, it was shown that P*ilvC* and P*sodA* showed strong expression in a GFP reporter assay with comparable expression levels as that of P*tac* in the case of P*ilvC* and around a 2.5-fold higher expression in the case of P*sodA* [65]. In the study presented here, expression levels of both promoters were lower than that of P*trc*. As discussed above, the promoter is one of several factors influencing reporter gene expression, making comparisons of reporter gene expression using different vector backbones, ribosome binding sites, etc. difficult. Nevertheless, the previously described vectors along with those described here allow for low, but constitutive expression and are of interest for the production of membrane proteins, transporter proteins, or enzymes that lead to the formation of toxic byproducts and for balancing of engineered pathways [48].

To maximize target gene expression, the design and usage of synthetic promoters for genetic engineering in *C. glutamicum* is on the rise, as these usually short promoters can achieve high transcription levels [37,38]. Moreover, the utilization of artificial promoters allows researchers to circumvent the challenges of regulatory interference in metabolic engineering approaches for overproduction. The P*H36* promoter was identified by Yim et al. 2013, where it showed a 60-fold higher expression level in the pCES plasmid background in comparison to that of the P*trc* promoter [37]. In contrast, here, P*H36* showed the weakest expression in the fluorescence assay. The synthetic P*syn* was designed and identified as it showed high promoter activity in a β-galactosidase assay [38]. Although this result is difficult to compare with the fluorescence assay used here, we have shown that the short synthetic promoter P*syn* (5′-TTGACATTAATTTGAATCTGTGTTATAATGGTTC-3′) enabled tunable and strong expression in combination with the *lac* operator sequence in *cis* (5′-CTGGAATTGTGAGCGGATAACAATTC-3′) and *lacIq* in *trans*. The newly constructed pVWEx6 vector showed a gene expression strength that is one magnitude higher than that of other commonly used expression vectors. Thus, the application of pVWEx6 can reduce the costs for IPTG, as 25 µM IPTG lead to comparable expression patterns as that of pVWEx1, pECXT99A, and pEKEx3 induced with 1 mM IPTG. Potentially, leakiness of these vectors can be further minimized as shown very recently for pEKEx2 by restoring *lacIq* gene sequences and by avoiding duplicate DNA sequences [67].

As an application case, we transferred the findings about elevated gene expression to fermentative sarcosine production with *C. glutamicum*. Strain SAR3* was based on the newly designed pECXT_P*syn* for expression of xylose utilization gene *xylAB* and it showed a volumetric productivity for sarcosine with 0.077 g L^−1^ h^−1^ as compared to that of the reference strain (0.051 g L^−1^ h^−1^) [11]. Our previous work on sarcosine production [11] identified slow growth with xylose as the limitation of the volumetric productivity for sarcosine. Here, we have shown that accelerating growth with xylose improved the volumetric productivity of SAR3* by 50% as compared to that of SAR3. We deliberately did not use pVWEx4 or pVWEx6 for *dpkA* expression here, since *N*-methylation of glyoxylate to yield sarcosine as catalyzed by DpkA does not limit sarcosine production. This was deduced from our previous work [68], where pVWEx1-*dpkA* supported a volumetric productivity of up to 0.35 g L^−1^ h^−1^ for *N-*methylation of pyruvate to *N*-methylalanine. Since it was shown that doubling of the MMA concentration as well as optimization of the carbon source composition (5 g/L xylose and 30 g/L actetate) improved the sarcosine production by SAR3 about 3-fold, combination of the newly constructed strain SAR3* with those culture conditions might further improve sarcosine production with *C. glutamicum*.

## Figures and Tables

**Table 1:** Strains and plasmids used in this study.

Strain	Characteristics of strains and plasmids	Reference
*C. glutamicum* strains		
WT	Wild type, ATCC 13032	[39]
SAR3	WT *ΔaceB icd^GTG^* (pVWEx1-*dpkA_*RBSopt) (pECXT99A-*xylAB*)	[11]
SAR3*	WT *ΔaceB icd^GTG^* (pVWEx1-*dpkA_*RBSopt) (pECXT-P*syn*-*xylAB*)	This work
Other strains		
*E. coli* DH5α	F-*thi-*1 *endA1 hsdr17*(r-, m-) *supE44* Δ*lacU169* (Φ80*lacZ*ΔM15) *recA1 gyrA96*	[40]
Plasmids		
pEKEx3	Spec^R^, *P_trc_lacI^q^*, pBL1 *oriV_Cg_*, *C. glutamicum*/*E. coli* expression shuttle vector	[41]
pEKEx3-*gfpUV*	pEKEx3 derivative for inducible expression of *gfpUV* from P*tac* promoter	[42]
pECXT99A (pECXT)	Tet^R^, *P_trc_lacI^q^*, pGA1 *oriV_Cg_*, *C. glutamicum*/*E. coli* expression shuttle vector	[2]
pECXT99A-*gfpUV*	pECXT99A derivative for inducible expression of *gfpUV* from P*trc* promoter	This work
pECXT99A-*xylAB*	pECXT99A derivative for inducible expression of *xylA* from *Xanthomonas campestris* and *xylB* from *C. glutamicum* from P*trc* promoter	[43]
pECXT-P*pgk*	pECXT99A derivative for constitutive expression from *C. glutamicum pgk* promoter	This work
pECXT-P*ilvC*	pECXT99A derivative for constitutive expression from *C. glutamicum ilvC* promoter	This work
pECXT-*PsodA*	pECXT99A derivative for constitutive expression from *C. glutamicum sodA* promoter	This work
pECXT-*PgapA*	pECXT99A derivative for constitutive expression from *C. glutamicum gapA* promoter	This work
pECXT-*Ptuf*	pECXT99A derivative for constitutive expression from *C. glutamicum tuf* promoter	This work
pECXT-*PH36*	pECXT99A derivative for constitutive expression from synthetic P*H36* promoter	This work
pECXT-*P45*	pECXT99A derivative for constitutive expression from synthetic P*45* promoter	This work
pECXT-*Psyn*	pECXT99A derivative for constitutive expression from synthetic P*syn* promoter	This work
pECXT-P*pgk*-*gfpUV*	pECXT99A derivative for constitutive expression of *gfpUV* from *C. glutamicum pgk* promoter	This work
pECXT-P*ilvC*-*gfpUV*	pECXT99A derivative for constitutive expression of *gfpUV* from *C. glutamicum ilvC* promoter	This work
pECXT-P*sodA*-*gfpUV*	pECXT99A derivative for constitutive expression of *gfpUV* from *C. glutamicum sodA* promoter	This work
pECXT-P*gapA*-*gfpUV*	pECXT99A derivative for constitutive expression of *gfpUV* from *C. glutamicum gapA* promoter	This work
pECXT-*Ptuf*-*gfpUV*	pECXT99A derivative for constitutive expression of *gfpUV* from *C. glutamicum tuf* promoter	This work
pECXT-*PH36-gfpUV*	pECXT99A derivative for constitutive expression of *gfpUV* from synthetic P*H36* promoter	This work
pECXT-P*45*-*gfpUV*	pECXT99A derivative for constitutive expression of *gfpUV* from synthetic P*45* promoter	This work
pECXT-P*syn*-*gfpUV*	pECXT99A derivative for constitutive expression of *gfpUV* from synthetic P*syn* promoter	This work
pECXT-P*syn-xylAB*	pECXT99A derivative for constitutive expression of *xylA* from *Xanthomonas campestris* and *xylB* from *C. glutamicum* from synthetic P*syn* promoter	This work
pVWEx1	Km^R^, *P_tac_lacI^q^*, pHM1519 *oriV_Cg_*, *C. glutamicum*/*E. coli* expression shuttle vector	[28]
pVWEx4	pVWEx1 derivative with mutation *repA*	This work
pVWEx6	pVWEx4 derivative with P*syn* promoter and lac operator for IPTG inducible expression	This work
pVWEx1-*gfpUV*	pVWEx1 derivative for IPTG-inducible expression of *gfpUV* from P*tac* promoter	[42]
pVWEx1-*dpkA*_RBS^opt^	pVWEx1 derivative for IPTG-inducible expression of *dpkA* from *P. putida* KT2440 and change of its start codon GTG to ATG and an RBS optimised for *C. glutamicum*	[11]
pVWEx4-*gfpUV*	pVWEx4 derivative for IPTG-inducible expression of *gfpUV* from P*tac* promoter	This work
pVWEx6-*gfpUV*	pVWEx6 derivative for IPTG-inducible expression of *gfpUV* from P*syn* promoter	This work

**Table 2:** Oligonucleotides used in this study.

Oligonucleotide	Target	Sequence (5′ → 3′)
HN12	Ptuf-fw	CTGTGCGGTATTTCACACCGCAGTTTTAGCGTGTCAGTAGGC
HN13	Ptuf-rv	CCGGGTACCGAGCTCGAATTCCATGTTACTGAATCCTAAGGGCAACG
HN14	PgapA-fw	CTGTGCGGTATTTCACACCGCAGTGTCTGTATGATTTTGCATCTG
HN15	PgapA-rv	CCGGGTACCGAGCTCGAATTCCATGCACGCACCAAACCTACTCACA
HN16	PilvC-fw	CTGTGCGGTATTTCACACCGCAATCCGGACAGATTGCACTCAAC
HN17	PilvC-rv	CCGGGTACCGAGCTCGAATTCCATGCATTATTGTTCTACCACACACATG
HN30	PsodA-fw	CTGTGCGGTATTTCACACCGCATACTTAGCTGCCAATTATTCCG
HN31	PsodA-rv	CCGGGTACCGAGCTCGAATTCCATGCCGCACCGAGCATATACATCT
HN97	Ppgk-fw	CTGTGCGGTATTTCACACCGCATAACGTGGGCGATCGATGC
HN98	Ppgk-rv	CCGGGTACCGAGCTCGAATTCCATGGCCGTACTCCTTGGAGATTTG
HA02	P45-fw	CTGTGCGGTATTTCACACCGCATTGGTCAGGGATTTTTTCCCG
HA03	P45-rv	CCGGGTACCGAGCTCGAATTCCATGGAACTTCTTCGTCACTTACTTTA
HA04	PH36-fw	CTGTGCGGTATTTCACACCGCACAAAAGCTGGGTACCTCTATCTG
HA05	PH36-rv	CCGGGTACCGAGCTCGAATTCCATGCATGCTACTCCTACCAACCAAG
HA06	Psyn-fw	GCGCCTGATGCGGTATTTTCTCCTTACGCATCTGTGCGGTATTTCACACCGCATTGACATTAATTTGAATCTGTGTTAT
HA07	Psyn-rv	CTGCAGGTCGACTCTAGAGGATCCCCGGGTACCGAGCTCGAATTCCATGGAACCATTATAACACAGATTCAAA
HA36	repA-fw	AAAATCGCTTGACCATTGCAGGTTG
HA37	repA-rv	CTTTAGCTTTCCTAGCTTGTCGTTGAC
HA40	repA-seq	TGCTCGTCAGACAGAGACGCAG
N101	pVWEx4-fw	ATGCATGCCGCTTCGCCTTCGATTGACATTAATTTGAATCTGTGTTATAATGGTTC
N102	pVWEx4-rv	CGGCCAGTGAATTCGAGCTCGAAATTGTTATCCGCTCACAATTCCAGGAACCATTATAACACAGATTCAA
N103	xylAB-fw	ATGGAATTCGAGCTCGGTACCCGGGGAAAGGAGGCCCTTCAGATGAGCAACACCGTTTTCATC
N104	xylAB-rv	CTGCAGGTCGACTCTAGAGGATCTTAGTACCAACCCTGCGTTGC
N105	Psyn-fw2	ATGCATGCCGCTTCGCCTTCGTTGACATTAATTTGAATCTGTGTTATAATGGTTC
N106	Psyn-rv2	GGCCAGTGAATTCGAGCTCGCTGCAGGTCGACTCTAGAGGATC
HN49	gfpUV-fw	ATGGAATTCGAGCTCGGTACCCGGGGAAAGGAGGCCCTTCAGATGAGTAAAGGAGAAGAACTTTTCA
HN50	gfpUV-rv	GCATGCCTGCAGGTCGACTCTAGAGGATCTTATTTGTAGAGCTCATCCATGC
582		ATCTTCTCTCATCCTCCA

**Table 3:** Comparison of *C. glutamicum* expression vectors. *: high-copy number variant of the pHM1519 replicon.

Plasmid	pEKEx3	pVWEx1	pVWEx4	pVWEx6	pECXT99A	pECXT_P*syn*
**Expression**	Inducible	Inducible	Inducible	Inducible	Inducible	Constitutive
**Repressor gene**	*lacI* ^q^	*lacI* ^q^	*lacI* ^q^	*lacI* ^q^	*lacI* ^q^	-
**Promoter**	P*tac*	P*tac*	P*tac*	P*syn*	P*trc*	P*syn*
**Induction factor**	6	76	121	51	40	non
**Maximal expression**	64	99	145	410	47	140
**Origin for *Cg***	pBL1	pHM1519	pHM1519 *	pHM1519 *	pGA1 mini	pGA1 mini
**Origin for *Ec***	ColE1 ori	ColE1 ori	ColE1 ori	ColE1 ori	pMB1	pMB1
**Resistance**	Spec	Kan	Kan	Kan	Tet	Tet

## Data Availability

The data presented in this study are available in article.
